# Neuroimaging and Neurocognitive Outcomes in Older Patients with Multiple Myeloma Treated with Chemotherapy and Autologous Stem Cell Transplantation

**DOI:** 10.21203/rs.3.rs-2733807/v1

**Published:** 2023-04-05

**Authors:** Denise D. Correa, Behroze A. Vachha, Raymond E. Baser, Adrian Koch, Phillip Wong, Suril Gohel, Sergio Giralt, James C. Root

**Affiliations:** Memorial Sloan Kettering Cancer Center; UMass Chan Medical School; Memorial Sloan Kettering Cancer Center; Memorial Sloan Kettering Cancer Center; Memorial Sloan Kettering Cancer Center; Rutgers University School of Health Professions; Memorial Sloan Kettering Cancer Center; Memorial Sloan Kettering Cancer Center

**Keywords:** Multiple myeloma, neurocognitive, MRI, resting state functional connectivity, cytokines

## Abstract

**Background:**

Many patients with hematological malignancies treated with stem cell transplantation (SCT) experience cognitive dysfunction. However, few studies have investigated treatment-related neurotoxicity in older adults with multiple myeloma (MM) treated with high dose chemotherapy (HDC) and autologous SCT (HDC/ASCT). In this study, we examined gray matter (GM) volume, resting state functional connectivity (RSFC), neurocognitive function (NF), and proinflammatory cytokines (PCy) in older patients with MM pre- and post-HDC/ASCT.

**Methods:**

Eighteen MM patients underwent magnetic resonance imaging, neurocognitive tests, and serum PCy measurement prior to HDC/ASCT, and fifteen patients completed follow ups an average of five months post-HDC/ASCT.

**Results:**

There were significant decreases in RSFC from pre- to post-HDC/ASCT in (1) the central executive network (CEN) involving the *left* dorsolateral prefrontal cortex and *right* posterior parietal cortex (p = 0.022), and (2) the CEN involving the *right* posterior parietal cortex and the salience network involving the *right* dorsal anterior cingulate cortex (p = 0.029); these comparisons were no longer significant after multiple comparisons correction. There were no significant changes in GM volumes or NF, except for improvement in attention (Digit Span Backward, p = 0.03). There were significant increases in several PCy post-HDC/ASCT (p ≤ 0.05).

**Conclusions:**

This pilot study showed decreased RSFC involving the left frontal, right posterior parietal and right anterior cingulate cortices in MM patients post-HDC/ASCT, relatively stable NF, and increases in PCy. These findings are congruent with studies in patients with hematological malignancies and other cancers and provide supporting evidence for the vulnerability of frontoparietal regions to chemotherapy adverse effects.

## Introduction

There is compelling evidence that chemotherapy is associated with neurotoxicity (Dietrich, Monje et al. 2008), with suggested mechanisms including demyelination, microglia activation, immune dysregulation, and stimulation of proinflammatory cytokines (PCy) (Ahles and Saykin 2007, Gibson and Monje 2021). Reductions in prefrontal gray matter (GM) volume (McDonald, Conroy et al. 2012) and changes in resting state functional connectivity (RSFC) (Kesler 2014, Vachha, Gohel et al. 2022) have been reported postchemotherapy in patients with breast and ovarian cancers. We reported reductions in prefrontal GM volume and changes in white matter (WM) integrity in patients with hematological malignancies treated with high dose chemotherapy (HDC) ± total body irradiation (TBI) and stem cell transplantation (SCT) (Correa, Root et al. 2013, Correa, Wang et al. 2016). Systematic reviews indicated that neurocognitive dysfunction is prevalent pre- and post-SCT (Buchbinder, Kelly et al. 2018).

Neurocognitive dysfunction has been documented in multiple myeloma (MM) patients after HDC and preautologous SCT (ASCT) with declines post-HDC/ASCT (Jones, Vichaya et al. 2013, Root, Campbell et al. 2020); however, other studies reported improvement in neurocognitive function (NF) months postchemotherapy (Jacobs, Small et al. 2007, Bury-Kaminska, Szudy-Szczyrek et al. 2021). Proinflammatory cytokines (PCy) play a critical role in tumor growth and progression in MM, with high levels identified in many patients (Musolino, Allegra et al. 2017). Aging can be associated with PCy elevations (Weiskopf, Weinberger et al. 2009), suggesting that older MM patients may be even more susceptible to cytokine dysregulation related to disease and treatment, which may contribute to neuroinflammation.

There is a paucity of research investigating neurotoxicity in *olderMM* patients undergoing HDC/ASCT, even though this intervention has been used more often in the elderly (Lemieux, Hulin et al. 2012), and NF has been recognized as a critical dimension of survivorship in older cancer patients (Mandelblatt, Jacobsen et al. 2014). In this pilot study, we assessed GM volume, RSFC, NF, and PCy in older MM patients prior to HDC/ASCT and an average of five months post-HDC/ASCT.

## Methods

### Patients

MM patients scheduled for conditioning HDC/ASCT were recruited through the Adult Bone Marrow Transplant Service at Memorial Sloan Kettering Cancer Center (MSK). Eligibility criteria: (1) MM diagnosis, (2) complete, partial, or very good partial disease remission at enrollment, as per standard International Myeloma Working Group Criteria, (3) age 60–75 at enrollment, (4) fluent in English. Exclusionary criteria: (1) disease progression during the study period, (2) CNS disease, or (3) history of neurological, psychiatric, or substance abuse disorders.

### Measures

#### Structural & Functional Imaging.

Patients were imaged in Tesla scanners (GE, Discovery 750W, USA with a GEM HNU 24-channel head coil) at MSK; five patients were imaged in two different Tesla scanners at each timepoint using the same parameters. *StructuralImaging:T1-weighted* anatomical images with whole brain coverage were obtained with spoiled gradient-recalled and high-resolution three-dimensional magnetization-prepared rapid acquisition with gradient-echo sequences. *Functional imaging*. For rsfMRI, T2*-weighted images were acquired with a single-shot gradient echo-planar imaging (EPI) sequence (TR/TE = 2500ms/30 ms, FA = 80°, slice thickness = 4 mm, matrix = 64 × 64). For the rsfMRI, patients were instructed to keep the eyes open and fixated on a crosshair.

##### Image Processing.

For *structural image processing*, VBM analysis was performed using the longitudinal processing stream in the VBM8 toolbox (http://dbm.neuro.uni-jena.de/vbm/) under the SPM8 software package (Version 8, Wellcome Department of Imaging Neuroscience, London, UK) within MATLAB (Version 7, Mathworks, Inc., Natick, MA). Following reconstruction, follow-up MPRAGE structural images were registered to baseline MPRAGE images for each subject, bias corrected, segmented into GM, WM, and cerebrospinal fluid compartments using the Montreal Neurologic Institute (MNI) T1 weighted template and tissue probability maps, linear and non-linear registered to MNI space, and the resulting GM tissue class smoothed using an isotropic Gaussian spatial filter (FWHM=8 mm). *For rsfMRI pre-processing*, a data pre-processing scheme was implemented according to published methods (Yang, Gohel et al. 2021, Vachha, Gohel et al. 2022). Briefly, the first five timepoints of fMRI data were removed to allow for T1 relaxation effects followed by head-motion correction, co-registration, segmentation, normalization to MNI standard space, temporal regression of 24 head-motion parameters (Friston, Williams et al. 1996), and five principal components of WM and cerebral spinal fluid time series (Behzadi, Restom et al. 2007), temporal filtering between 0.01 to 0.1 Hz, and spatial-smoothing with 6 mm full-width-at-half-maximum Gaussian filter. Using the head motion parameters, we calculated subject-specific measures of mean frame-wise displacement (Jenkinson, Bannister et al. 2002).

#### Neurocognitive Tests & Self-Report Scales.

Patients completed neurocognitive tests in domains sensitive to cancer therapy adverse effects, (Wefel, Vardy et al. 2011) and mood/fatigue self-report scales on the same day or within two weeks of the MRIs.

##### Attention and Working Memory.

Longest Digit Span Forward-LDSF; Longest Digit Span Backward-LDSB; Longest Number Sequencing-LSS (WAIS-IV) (Wechsler 2008); Brief Test of Attention (BTA) (Schretlen, Bobholz et al. 1996); Auditory Consonant Trigrams Test (ACT). (Shura, Rowland et al. 2016)

##### Executive Functions.

Trail Making Test Parts A & B (TMTA & TMTB) (Reitan R 1985); Controlled Oral Word Association Test (COWA). (Benton and Hamscher 1989)

##### Verbal Memory.

Hopkins Verbal Learning Test-Revised: Total, Delayed Recall, Discrimination Index (HVLT-R-T; HVLT-R-D; HVLT-R-DI). (Bennedict, Schretlen et al. 1998).

##### Self-Report Scales.

Center for Epidemiological Study-Depression (CES-D) (Radloff 1977);

Functional Assessment of Chronic Illness Therapy-Fatigue Subscale, Version 4 (FACIT-FS V-4). (Cella 1997).

#### Multiplex Cytokine Panel.

Blood samples were collected pre- and post-HDC/ASCT on the same day as the neurocognitive assessment and delivered to the Immune Monitoring Core Facility at MSK for plasma isolation and frozen storage until ready for batch analysis. PCy were quantitated from thawed plasma samples following manufacturer instructions for the V-PLEX Human Proinflammatory Panel 10-plex kit (Meso Scale Diagnostics-MSD, Cat #K15049D-1), which included interleukin Ibeta (IL-1 β), interleukin 2 (IL-2), interleukin 4 (IL-4), interleukin 6 (IL-6), interleukin 8 (IL-8), interleukin 10 (IL-10), interleukin 12 (IL-12), interleukin 13 (IL-13), interferon gamma (IFNγ), and tumor necrosis factor alpha (TNF-α).

### Statistical and Imaging Analyses

#### Voxel-Based Morphometry (VBM).

Following omnibus testing, pairwise t-tests were performed at the group level to analyze within group changes from pre- to post-HDC/ASCT. For the structural contrast, initial uncorrected voxel-wise threshold was p≤ 0.001 with resulting maps family-wise error corrected over the whole brain at p≤0.05.

##### Resting State Functional Connectivity Analysis (RSFC)

Resting State Functional Connectivity Analysis (RSFC) was performed using region-of-interest based correlation as described previously (Biswal, Yetkin et al. 1995). Three resting state networks (RSNs) were extracted for prioritized analyses: the central executive network (CEN), the salience network (SN), and the default mode network (DMN). Spherical regions of interest (ROIs) were created surrounding the ROI coordinates appropriate to the RSNs of interest. The CEN and SN ROIs were created using the coordinates defined by Uddin et al. (Uddin, Supekar et al. 2011) and Yang et al. (Yang, Gohel et al. 2021), respectively. The DMN ROIs were created using NeuroSynth (Yarkoni, Poldrack et al. 2011) with the functional connectivity and co-activation map derived in 02/2022 using the term “default mode”. For each of the coordinates, a 6mm spherical ROI was created. Table 1 lists the MNI coordinates for each ROI.

Correlation matrices were produced by extracting the time course from each of the ROIs and computing the Pearson correlation coefficient *(r)* between each ROI pair in the CEN, SN, and DMN. Each of the pairwise ROI correlations were Fisher z-transformed for further statistical analysis. Changes in RSFC z-scores from pre- to post-HDC/ASCT were assessed using linear mixed models, adjusting for scanner (1, 2).

#### Neurocognitive Analysis.

Raw neurocognitive test scores were transformed into z-scores based on age-adjusted normative values. Neurocognitive test z-scores and self-report scale scores were summarized at each timepoint using descriptive statistics, and differences in scores from pre- to post-HDC/ASCT were compared using Wilcoxon signed rank tests. Standardized effect sizes (i.e., Cohen’s *d)* were calculated to quantify the magnitude of score changes over time. False discovery rate (FDR) was used to adjust p-values for multiple comparisons. The Reliable Change Index (RCI), which represents the change in scores divided by the standard error of measurement, was used to identify patients whose raw scores improved or declined beyond expected due to practice effects and measurement error. For each test score, we calculated the proportion of patients with RCI-indicated reliable decline.

### Cytokine Panel Analysis

Cytokine data was analyzed using the MSD Discovery Workbench^ò^ software to measure levels of a ten-PCy panel at each timepoint. A four-parameter logistic (4PL) fit calibration curve was generated for each analyte using the standards to calculate the concentration of each analyte. Upper and lower limits of quantitation for each PCy were established as the highest and lowest points of the standard curve on each plate whose back calculated values were within 80–120% of the expected values of the 4PL regression fit and exhibited less than 20% CV across the duplicate standard wells.

#### Correlations.

Spearman correlations were calculated to assess the association of RSN z-scores, neurocognitive test z-scores, self-report scale scores, and PCy levels separately for each timepoint.

## Results

Eighteen MM patients completed a neurocognitive assessment and a brain MRI pre-HDC/ASCT, and fifteen patients were available for follow up an average of five months (median = 5.82, range = 3.50 −7.00) post-HDC/ASCT. One patient was excluded from the imaging analysis due to scan misregistration at follow up. Thirteen patients provided blood samples for PCy analysis at each timepoint. Descriptive statistics for demographic and disease variables are presented in Table 2. All patients received conditioning HDC the day before or the same day as the ASCT. Five patients were treated with Siltuximab, an IL-6 blocker (median half-life elimination: ~21 days, range:14 to 30 days) (Kurzrock, Voorhees et al. 2013, Chen, Teachey et al. 2016), infused seven days pre-HDC/ASCT and twenty-one days post-HDC/ASCT as part of a separate MSK protocol, which did not require high IL-6 levels for enrollment. In this study, data collection occurred prior to the first Siltuximab infusion and at least 3–4 months after the second infusion for all patients. Among the three patients who did not return for follow-up, one had disease progression, one was deceased, and one relocated.

### Structural & Functional Imaging

**Figure 1:**
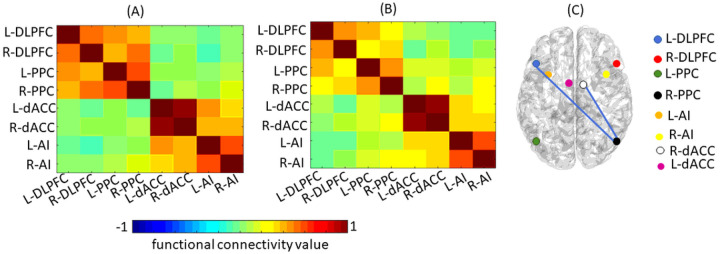
**(A)** Mean Functional Connectivity for Central Executive Network and Salience Network pre-ASCT. **(B)** Mean Functional Connectivity for Central Executive Network and Salience Network post-ASCT **(C)** Group level difference in Functional Connectivity between pre- and post-ASCT. Blue lines represent significantly decreased connectivity (p<0.05) from pre- to post-ASCT. DLPFC= Dorsolateral Prefrontal Cortex, PPC= Posterior Parietal Cortex, dACC=Dorsal Anterior Cingulate Cortex, AI=Anterior Insula; L=Left, R=Right.

The results showed significant decreases in RSFC in (1) CEN ROIs involving the *left* dorsolateral prefrontal cortex (L-DLPFC) and *right* posterior parietal cortex (R-PPC) (p=0.022), and (2) the CEN ROI involving the R-PPC and the SN ROI involving the *right* dorsal anterior cingulate cortex (R-dACC) (p=0.029) from pre- to post-HDC/ASCT (Figure 1); these comparisons were no longer significant after correction for multiple comparisons. There were no significant changes in RSFC in the DMN. VBM analysis results showed no significant changes in regional GM volumes (FWE corrected, p>0.05).

### Neurocognitive Function & Self-Report Scales

The neurocognitive tests z-scores and self-report scales scores are presented in Table 3. Mean z-scores were within the average range for all tests, except for ACT-perseverations. There were no significant changes in from pre- to post-HDC/ASCT, except for a significant improvement in attention (Longest Digit Span Backward, p=0.03); this comparison was no longer significant after multiple comparisons correction. There were no significant changes in the CES-D and FACIT-FS scores, and scores were within normal limits at each timepoint (Radloff 1977, Webster, Cella et al. 2003). There were no significant differences on the neurocognitive tests between the fifteen patients who completed both timepoints and the three patients who performed the pre-HDC/ASCT assessment only.

The RCI results showed that most patients (>67%) had no reliable change on the neurocognitive tests from pre- to post-HDC/ASCT. Patients had reliable declines in the HVLT-R-D (7%), TMT A (34%), TMT B (13%), and BTA (7%). Reliable improvements were seen in the HVLT-R-T (13%), HVLT-R-D (7%), TMT A (34%), TMT B (20%), and BTA (27%).

### Multiplex Cytokine Panel

PCy levels were generally low in most patients at both timepoints, and pre-HDC/ASCT values were below the limits of quantitation for IL-1 b, IL-2, IL-4, IL-10, IL-12, and IL-13. There were significant increases in PCy post-HDC/ASCT, including IL-1 b, IL-2, IL-4, IL-8, IL-12, IL-13, and TNFa (p< 0.05, FDR corrected). Table 4 includes PCy medians and ranges. The five patients treated with Siltuximab exhibited high IL-6 levels post-HDC/ASCT and showed increases in IL-1 b, IL-2, IL-4, IL-12, and IL-13 levels, but the comparisons were not statistically significant. There were no significant differences in the neurocognitive tests or self-report scales between patients with high versus normal IL-6 levels post-HDC/ASCT.

#### Correlations.

There were no significant correlations among RSN z-scores, neurocognitive test z-scores, self-report scale raw scores, and PCy levels either pre- or post-HDC/ASCT.

## Discussion

This is the first study describing alterations in RSFC in older MM patients treated with HDC/ASCT. The results showed decreased connectivity in the CEN involving the L-DLPF and R-PPC and in the CEN R-PPC and the SN R-dACC from pre- to an average of five months post-HDC/ASCT, suggesting that adverse effects of HDC may be of concern in this population. The CEN has been described as a frontoparietal network involved in attention control, working memory, and processing speed (Seeley, Menon et al. 2007, Vincent, Kahn et al. 2008, Nee and D’Esposito 2017). The SN includes the dACC and anterior insula (AI) cortex and is involved in monitoring and processing of errors and conflict (Seeley, Menon et al. 2007, Vincent, Kahn et al. 2008, Menon and Uddin 2010) (Heilbronner and Hayden 2016), and attention control in the presence of distraction (Shenhav, Botvinick et al. 2013). Although we found no significant changes in the DMN, the CEN and SE results are overall consistent with breast and ovarian cancer studies suggesting that frontoparietal regions are susceptible to the adverse effects of chemotherapy (Kesler 2014, Feng, Wang et al. 2020, Shen, Tsai et al. 2021) (Correa, Root et al. 2017) (Vachha, Gohel et al. 2022).

A structural neuroimaging literature review in non-CNS cancer patients (McDonald 2021) described consistent findings of reduced GM volume mostly in frontal, temporal and parietal areas, and diffuse alterations in WM integrity, mostly in patients treated with chemotherapy. Our study in patients with hematologic malignancies treated with HDC ± TBI and SCT (Correa, Root et al. 2013) showed GM volume reductions in the bilateral middle frontal gyrus from pre- to one-year post-SCT. However, the current results showed no significant changes in regional GM volume post-HDC/ASCT, suggesting no significant adverse effects on brain structure.

It is estimated that at least 50% of patients with hematological malignancies experience neurocognitive dysfunction prior to SCT, with either stable performance or declines months to years post-SCT (Syrjala, Dikmen et al. 2004, Jacobs, Small et al. 2007, Harrison, Sharafeldin et al. 2021). In this study, NF was within the average range, although below expected levels considering the mean education of the cohort, with impairment on a test of susceptibility to interference/working memory. This could be in part related to the disease and residual adverse effects of proteasome inhibitors, chemotherapy, and immunotherapy, (Rollin-Sillaire, Delbeuck et al. 2013) and in some patients, to the side effects of dexamethasone (Prado and Crowe 2019) and lenalidomide (Calvi, Marchetti et al. 2019). NF remained relatively stable, with improvement in attention, suggesting no significant adverse effects from HDC/ASCT; however, it has been suggested that stable NF post-SCT may represent lack of improvement expected due to practice effects (Phillips, McGinty et al. 2013). Self-report scales indicated no depression or fatigue, and no changes from pre- to post-HDC/ASCT. Some studies reported improvement in NF following chemotherapy in older MM patients (Bury-Kaminska, 2021), and one-year post-SCT in a sample of mostly MM patients. (Jacobs et al. 2007) However, neurocognitive dysfunction has been reported in MM patients after HDC and pre-ASCT, with declines one and three months post-HDC/ASCT (Jones, Vichaya et al. 2013).

Cytokine dysregulation is common in MM and may influence the development of adverse effects (Musolino, Allegra et al. 2017). High PCy levels may interfere the blood brain barrier integrity (Wardill, Mander et al. 2016), and induce proliferation of microglia and CNS inflammation (Streit, Hurley et al. 2000, Bachiller, Jimenez-Ferrer et al. 2018). Increased PCy levels have been associated with neurocognitive dysfunction (Cheung, Ng et al. 2015, Henneghan, Palesh et al. 2018) and with changes in brain structure (Kesler, Janelsins et al. 2013) in breast cancer patients. Neurocognitive dysfunction was associated with high IL-6 levels in patients with leukemia and myelodysplastic syndrome (Meyers, Albitar et al. 2005) (Hoogland, Nelson et al. 2019). However, the mechanisms of chemotherapy-related PCy dysregulation and NF are not well understood, with conflicting results on the strength and direction of these associations (Janelsins, Mustian et al. 2012, Harrison, Sharafeldin et al. 2021). We observed low PCy levels at both timepoints, with significant increases post-HDC/ASCT, possibly influenced by HDC adverse effects (Condomines, Veyrune et al. 2010). In some patients, the effects of dexamethasone and lenalidomide may have also influence PCy levels. In the patients treated with Siltuximab, IL-6 and other PCy levels increased about five months post-HDC/ASCT. There is evidence that IL-6 blockade may result in a paradoxical increase in IL-6 and systemic inflammation. (Speake, Habib et al. 2022) (Kostek, Nagaraju et al. 2012). However, additional research would be required to reconcile our findings with the role of MM and its treatment on PCy dysregulation and neurotoxicity.

In this pilot study, changes in RSFC were more pronounced than in NF, possibly reflecting the use of compensatory mechanisms to maintain cognitive performance in the context of decreased CEN and SE connectivity (McDonald, Conroy et al. 2012), and the greater sensitivity of advanced neuroimaging tools to detect subtle alterations in functional connectivity in vulnerable regions. However, the RSFC results were no longer significant after multiple comparisons correction, and there were no significant associations between NF, RSFC, and PCy levels. These findings may be in part related to reduced power to detect small changes and associations beyond what was reported, and to MM patients receiving fewer lines of prior chemotherapy and less neurotoxic agents and conditioning regimens, compared to other hematologic malignancies (Phillips, McGinty et al. 2013).

## Conclusion

Decreased RSFC in older MM patients following HDC/ASCT provides further evidence for the prevailing notion that frontal-parietal regions may be vulnerable to chemotherapy adverse effects. Longitudinal studies with larger sample sizes are needed to further investigate the neural correlates of chemotherapy-related neurotoxicity and the role of PCy in older MM patients, with the goal of developing targeted therapeutic interventions.

## Figures and Tables

**Table 1.** MNI Coordinates

Network Name	Region Name	X	Y	Z
**Default Mode Network**				
	Posterior Cingulate Cortex	−2	−54	26
	Medial Prefrontal Cortex	2	50	−6
	L-Angular Gyrus	−50	−62	32
	R-Angular Gyrus	46	−70	32
**Salience Network**				
	L-Dorsal Anterior Cingulate Cortex	−5	26	31
	R-Dorsal Anterior Cingulate Cortex	5	26	31
	L-Anterior Insula	−34	15	−4
	R-Anterior Insula	37	20	−6
**Central Executive Network**				
	L-Dorsolateral Prefrontal cortex	−46	20	44
	R-Dorsolateral Prefrontal cortex	46	20	44
	L-Posterior Parietal Cortex	−40	−56	44
	R-Posterior Parietal Cortex	52	−52	50

L=Left; R=Right.

**Table 2.** Demographic Characteristics & Treatment History (N=18)

**Demographics**	
Sex (M/F)	10/8
Handedness (R/L)	17/1
Education (years)	
Mean (SD)	14.33 (3.58)
Median (Range)	14.5 (13–18)
Age at study entry (Years)	
Mean (SD)	66.11 (3.60)
**Treatment regimen pre-ASCT**	
RVd	5 (28%)
KRd	5 (28%)
Lenalidomide	4 (22%)
KRd + RVd	1 (5.5%)
CyBorD ± KRd	2 (11 %)
CyBordD + KRd + RVd	1 (5.5%)
**Response to pre-ASCT treatment**	
CR	7 (39%)
VGPR	9 (50%)
PR	2 (11 %)
**Time since pre-ASCT treatment**	
0 – 1 months	11 (61%)
2 months	5 (28%)
>2 months	2 (11 %)
**ASCT Conditioning Regimen**	
Melphalan - Single Dose	16 (89%)
Melphalan - Multiple Dose	2 (11 %)
**Time from Baseline to ASCT**	
≤ 1 month	15 (83%)
1.1 – 4 months	3 (17%)
**Relevant Medications**	
Pre-ASCT	
Dexamethasone	6 (33%)
Lenalidomide	4 (22%)
Post-ASCT	
Dexamethasone	0 (0%)
Lenalidomide	3 (17%)

SD= standard deviation, CR= complete response, VGPR=very good partial response

PR=partial response; KRd= carfilzomib/lenalidomide/dexamethasone,

RVd=lenalidomide/bortezomib/dexamethasone,

CyBorD=cyclophosphamide/bortezomib/dexamethasone

**Table 3.** Neurocognitive Test Z-Scores & Self-Report Scales Raw Scores

Measures	Pre-ASCT N=15 Mean (SD)	Post-ASCT N=15 Mean (SD)
*Attention/Working Memory*		
LDSF	0.16 (1.18)	0.20 (1.21)
LDSB	−0.02 (0.96)	0.50 (1.09)*
LLSS	−0.14 (1.05)	0.17 (1.14)
BTA	−0.07 (1.07)	0.33 (0.97)
ACT-T	−0.24 (1.02)	−0.46 (1.10)
ACT-P	−1.60 (1.41)	−1.64 (1.55)
*Executive Functions*		
TMT A	−0.35 (0.80)	−0.05 (1.01)
TMT B	−0.71 (1.03)	−0.16 (1.13)
COWA	−0.94 (1.17)	−0.51 (1.08)
*Verbal Memory*		
HVLT-R-T	−0.63 (1.09)	−0.49 (0.93)
HVLT-R-D	−0.58 (0.98)	−0.55 (1.42)
HVLT-R-DI	−0.02 (0.86)	0.34 (0.69)
*Self-Report Scales*		
CES-D	9.67 (5.49)	9.73 (6.37)
FACIT-FS	38.89 (7.55)	39.40 (6.72)

LDSF= Longest Digit Span Forward; LDSB= Longest Digit Span Backward; LNS = Longest Number Sequencing; BTA= Brief Test of Attention; ACT-T= Auditory Consonant Trigrams - Total Score; ACT-P = Auditory Consonant Trigrams - Perseverations; TMTA= Trail Making Test A; TMTB= Trail Making Test B; COWA= Controlled Oral Word Association Test; HVLT-R-T= Hopkins Verbal Learning Test-Revised-Total Learning; HVLT-R-D= Hopkins Verbal Learning Test-Revised-Delay; HVLT-R-DI= Hopkins Verbal Learning Test-Revised-Discrimination Index; CES-D= Center for Epidemiological Study-Depression; FACIT-FS= Functional Assessment of Chronic Illness Therapy Version IV-Fatigue Subscale.

* p=0.03.

**Table 4.** Cytokine Levels (pg/ml)

Cytokines	Pre-ASCT N=13 Median (range)	Post-ASCT N=13 Median (range)
IL-1 β	0.00 (0.00–0.00)	0.00 (0.00–0.18)*
IL-2	0.08 (0.01–0.28)	0.35 (0.19–1.05)*
IL-4	0.01 (0.00–0.02)	0.01 (0.00–2.53)*
IL-6	0.78 (0.65–0.86)	2.30 (0.58–1,572)
IL-8	4.53 (3.41–6.62)	7.70 (6.75–10.96)*
IL-10	0.19 (0.12–0.42)	0.28 (0.15–0.48)
IL-12	0.05 (0.01–0.14)	0.10 (0.08–3.03)*
IL-13	0.00 (0.00–0.13)	0.25 (0.00–0.88)*
IFNγ	2.39 (1.82–6.91)	4.15 (2.43–7.38)
TNFα	0.56 (0.44–0.75)	0.94 (0.60–1.04)*

Pg/ml=picograms per milliliter; IL-1 β = interleukin 1 beta, IL-2 = interleukin 2, IL-4 = interleukin 4, IL-6 = interleukin 6, IL-8 = interleukin 8, IL-10 = interleukin 10, IL-12 = interleukin 12, IL-13 = interleukin 13, IFNy = interferon gamma, TNF-α = tumor necrosis factor alpha.

* p< 0.05 (FDR corrected).

## Data Availability

Data will be available upon request to the corresponding author.
